# Case of Fibro-cystic Tumor of the Breast

**Published:** 1878-04

**Authors:** P. S. Hayes


### CASE OF FIBRO-CYSTIC TUMOR OF THE BREAST.

By Prof. P. S. Hayes.

I was consulted Sept. 18, 1877, by Mrs. D., aged 47, who ten years previously had noticed a tumor as large as a pea at the outer side of the left nipple. It had increased in size slowly but continuously ever since, and was at the time of examination much larger than the mammary gland itself. It had never been painful, nor had it caused other discomfort than that produced by its weight; the growth of the tumor had not been retarded by the menopause, which had been passed some years previously. There were also two tumors, one on either side of the neck just above the clavicle. That on the left side had appeared simultaneously with that in the breast, had gradually enlarged, but had ceased growing several years previously. The pectoral tumor was quite movable, the nipple flattened but not retracted; the skin appeared normal except that in the region of the nipple it was of a bluish tint, due to the presence of dilated capillaries, and was here adherent to the tumor beneath. The breast was firm and elastic and contained circumscribed areas where fluctuation was more or less distinct. There were no enlarged glands in the axilla. The growths were pronounced non-malignant fibro-cystic tumors.

Sept. 20 I removed the largest tumor, making a triangular flap whose apex was at the axilla and base at the sternum. The growth was easily removed, there being no adhesions. The patient made a rapid recovery. The tumor weighed 4¼ pounds; was 8½ inches long, 6 inches broad, and 4 thick. It contained numerous cysts, the contents of which varied in color from yellowish-white to reddish-brown, and in consistence from serum to white of egg.

*Microscopy.*—A thin section of the tumor removed by Dr. Hayes shows the structure of "Adeno-Fibroma." The connective tissue of the interlobular spaces is markedly increased; and this increase is made up of a mixture of round cells, spindle cells, and what may very properly be called nucleated fibers. These three structural elements represent as many stages of differentiation of the same cells; and, so far as the connective tissue is concerned, its abnormality consists in the presence of an undue amount of undeveloped or half-grown cells; in other words, of cells which multiply so rapidly that very many of them never reach maturity. The lobules (acini) are distended with half-grown epithelial cells, and from many of them diverticula or infundibula have developed, these diverticula being also crowded with proliferating epithelial cells. But the two classes of proliferating cells—that is, connective tissue cells and proper gland cells—retain their typical forms. They grow and multiply in accordance with their recognized law of growth. Hence this great mass of distended lobules and connective tissue cannot properly be regarded as a malignant growth. But so nearly is it allied to the malignant tumors, that if removed, it is more than likely to recur, not as an adenoma, but as a true carcinoma, presenting a veritable atypical cell growth. I. N. D.

